# TNF Superfamily: A Growing Saga of Kidney Injury Modulators

**DOI:** 10.1155/2010/182958

**Published:** 2010-10-04

**Authors:** Maria D. Sanchez-Niño, Alberto Benito-Martin, Sara Gonçalves, Ana B. Sanz, Alvaro C. Ucero, Maria C. Izquierdo, Adrian M. Ramos, Sergio Berzal, Rafael Selgas, Marta Ruiz-Ortega, Jesus Egido, Alberto Ortiz

**Affiliations:** ^1^IIS- Fundación Jiménez Díaz, 28040 Madrid, Spain; ^2^Nefrologia e Transplantação Renal, Hospital de Santa Maria, CHLN, EPE, 1600 Lisbon, Portugal; ^3^Servicio de Nefrologia, Fundacion para la Investigacion Biomedica del Hospital Universitario La Paz, RedinREN, Instituto de Salud Carlos III, Fondos FEDER, 28046 Madrid, Spain; ^4^Universidad Autónoma de Madrid, Madrid, Spain; ^5^Fundación Renal íñigo Álvarez de Toledo, Madrid, Spain; ^6^Unidad de Diálisis, Fundación Jiménez Díaz, Av Reyes Católicos 2, 28040 Madrid, Spain

## Abstract

Members of the TNF superfamily participate in kidney disease. Tumor necrosis factor (TNF) and Fas ligand regulate renal cell survival and inflammation, and therapeutic targeting improves the outcome of experimental renal injury. TNF-related apoptosis-inducing ligand (TRAIL and its potential decoy receptor osteoprotegerin are the two most upregulated death-related genes in human diabetic nephropathy. TRAIL activates NF-kappaB in tubular cells and promotes apoptosis in tubular cells and podocytes, especially in a high-glucose environment. By contrast, osteoprotegerin plays a protective role against TRAIL-induced apoptosis. Another family member, TNF-like weak inducer of apoptosis (TWEAK induces inflammation and tubular cell death or proliferation, depending on the microenvironment. While TNF only activates canonical NF-kappaB signaling, TWEAK promotes both canonical and noncanonical NF-kappaB activation in tubular cells, regulating different inflammatory responses. TWEAK promotes the secretion of MCP-1 and RANTES through NF-kappaB RelA-containing complexes and upregulates CCl21 and CCL19 expression through NF-kappaB inducing kinase (NIK-) dependent RelB/NF-kappaB2 complexes. In vivo TWEAK promotes postnephrectomy compensatory renal cell proliferation in a noninflammatory milieu. However, in the inflammatory milieu of acute kidney injury, TWEAK promotes tubular cell death and inflammation. Therapeutic targeting of TNF superfamily cytokines, including multipronged approaches targeting several cytokines should be further explored.

## 1. TNF Superfamily

Tumor necrosis factor (TNF) was isolated and cloned 25 years ago [1, 2]. This molecule became the prototype of a growing familyof related proteins called the TNF superfamily (TNFSF) that share common features. Most members of the family are synthesized as type II transmembrane proteins and share a common structural motif, the TNF homology domain (THD), that mediates self-trimerization and receptor binding [3, 4]. The extracellular domain can be cleaved by specific proteases to generate soluble cytokines. 

The TNF receptor superfamily (TNFRSF) includes receptors for the TNFSF ligands. Most are type I transmembrane glycoproteins and are characterized by the presence of extracellular cysteine-rich domains [5]. TNFRSF proteins are usually membrane bound, but some also exhibit a soluble form [6]. Similarly to TNFSF ligands, the functional receptors are usually trimeric. Ligands and receptors undergo clustering during signal transduction [7, 8].

Most TNFSF ligands bind to a single receptor; some bind to more than one, and there is evidence of crosstalk between receptors for different ligands [5]. Genetic approaches have defined the physiological function linked to the individual ligands or receptors [9].

Ligand activation of TNFRSF members modulates cell proliferation, survival, differentiation, and apoptosis [9]. Such cellular events participate in a broad array of biological processes such as inflammation, fibrosis, the immune response, and tissue repair [10]. TNFSF and TNFRSF proteins have been targeted therapeutically, and several drugs and biologicals are approved for use in inflammatory and autoimmune diseases [11]. Cumulative experimental evidence supports a role of the TNFSF/TNFRSF members in kidney injury outlined in Table 1. 

Many TNFSF cytokines, including TNF, FasL, TRAIL, and TWEAK may activate the NF-kappaB family of transcription factors [12]. However, different cytokines activate different members of the NF-kappaB family. NF-kappaB DNA-binding complexes are homo- or hetero-dimers of five Rel proteins: NF-kappaB1 (p50, generated from p105), NF-kappaB2 (p52, generated from p100), RelA (p65), RelB, and c-Rel. The nuclear translocation and DNA binding of NF-kappaB occurs by two main pathways. Classical or canonical NF-kappaB activation is a rapid and transient response to a wide range of stimuli whose main effector is RelA/p50. The alternative or noncanonical NF-kappaB pathway is a more delayed response to a smaller range of stimuli resulting in NIK activation and DNA binding of RelB/p52 complexes. There is evidence that these pathways target a partially overlapping set of inflammatory mediators. NF-kappaB also regulates cell proliferation, survival, and differentiation.

TNFSF/TNFRSF members mediate different functions, in different tissues that depend on the surrounding milieu. Unraveling their complex and pleiotropic actions will be essential for their use as therapeutic targets.

## 2. TNF and Kidney Injury

TNF (TNFSF2) was the first member of the family to be implicated in the pathogenesis of kidney injury [13]. TNF is a potent proinflammatory cytokine and an important mediator of inflammatory tissue damage. TNF also has an immunoregulatory role [11].

In the kidney, TNF is expressed, synthesized, and released by infiltrating macrophages and by intrinsic kidney cells, namely, endothelial, mesangial, glomerular, and tubular epithelial cells [14]. In vivo, the TNF expression pattern seems to be related to the primary kidney compartment injured [15]. TNF activates two receptors, TNFR1 and TNFR2. TNFR1 is present in normal glomeruli and is upregulated on infiltrating leukocytes in response to renal injury. TNFR2 is usually not expressed in normal kidney and is upregulated in tubular cells in response to renal injury [15].

These receptors induce different and possibly opposing functions in inflammation and immunity, and the differential contribution of TNFR1- and TNFR2-mediated TNF signaling in renal lesions has only recently started to be explored [11, 16].

Increasing evidence has implicated TNF as a major participant in the pathogenesis of kidney injury, promoting inflammation, apoptosis, and accumulation of extracellular matrix, reducing glomerular blood flow and damaging the glomerular permeability barrier with development of albuminuria [14, 17–22]. The pathogenic role of TNF as well as the potential benefits of modulating TNF activity has been shown in models of immune complex-mediated glomerulonephritis, lupus nephritis, antineutrophil cytoplasmic antibodies (ANCA-) associated glomerulonephritis, minimal change disease, diabetic nephropathy (DN), acute kidney injury (AKI), obstructive uropathy, and kidney allograft rejection [14, 15, 19, 21, 23–26]. TNFR1 or TNFR2 deficiency protects mice from cisplatin-induced AKI [27, 28] and obstructive uropathy [29].

However, TNF also has immunosuppressive functions, depending on the surrounding milieu, the timing of the inflammatory response, and the differential interaction with its receptors [15]. Thus, TNFR1 deficiency enhances disease in MRL-lpr/lpr lupus mice [30], while TNFR2 deficiency confers protection from autoimmune renal injury [31, 32].

In 1995, we wrote “First candidates for (anti-TNF strategies) trials will be …. rapidly progressive glomerulonephritis and vasculitis” [14]. In 2010, emerging clinical data suggest a potential benefit of TNF antagonism in lupus nephritis [33, 34] and Wegener's granulomatosis [35, 36]. However, overall experience with different TNF formulations in vasculitis is inconclusive, and questions remain on the optimal combination of immunosuppressive drugs and specific subgroups of patients that might benefit [37–40]. Moreover, TNF blockade has been associated with the emergence of autoantibodies [41] and lupus syndromes [41, 42] and with the development of infection, particularly reactivation of tuberculosis [43, 44]. The net effect of TNF actions depends on the balance between the proinflammatory and immunosuppressive functions, and current efforts are focusing on the selective inhibition of its deleterious actions.

## 3. Fas Ligand: A New Kid in the Block

Fas (Apo-1/CD95/TNFRSF6) is a 45-kDa type I membrane receptor containing an intracellular death domain (DD). Fas is engaged by Fas ligand (FasL/TNFSF6), a 36–40-kDa type II membrane TNFSF member [45]. The regulation of Fas/FasL functions is complex. Metalloprotease-mediated soluble FasL (sFasL) shedding from membrane-bound FasL (mFasL) as well as decoy receptors modulates the system [46–48]. Thus, mFasL induces apoptosis more efficiently than sFasL [49, 50].

Fas activation triggers apoptosis through recruitment and activation of caspase-8 by the adaptor protein, Fas-associated protein with dead domain (FADD) [51]. Nonapoptotic effects, such as proliferation, cell differentiation and inflammation, are also triggered in a range of cell types [51–53].

FasL and Fas play a critical role in modulating the immune response, including the peripheral deletion of autoimmune cells, activation-induced T cell death, and T cell-mediated cytotoxicity [45], thereby guarding against autoimmunity and tumor development [51].

The Fas receptor is constitutively expressed by mesangial and tubular cells, podocytes, and fibroblasts and is upregulated by noxious stimulus and inflammation [54–57]. Several inflammatory cytokines and nephrotoxins upregulate tubular cell Fas [58–61]. Potential sources of renal FasL include infiltrating leukocytes and intrinsic renal cells, mainly tubular, but also mesangial, endothelial, and fibroblastic cells [54]. FasL is normally expressed by renal cells and is upregulated during renal injury [62]. Activation of NF-kappaB upregulates FasL in cultured mesangial cells exposed to inflammatory mediators [63] and in HIV-associated nephropathy podocytes [55]. Fas and FasL are segregated from each other to different cellular compartments in kidney tubular cells: Fas is restricted to the basolateral surface, while FasL is sequestered to an intracellular compartment and, to a lesser extent, the apical surface [64]. This segregation may prevent autocrine/paracrine cell death, but is lost upon disruption of tight junctions by physical injury, ischemia, or proinflammatory cytokines [64].

**Figure 1:**
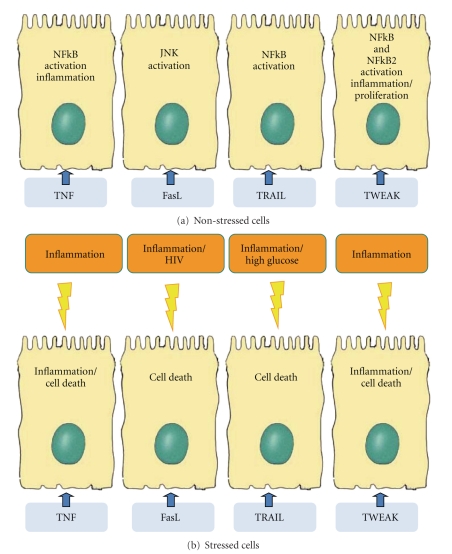
Schematic representation of TNFSF cytokine actions on tubular renal cells. The microenvironment influences the cell response. Among the many potential microenvironmental factors, we have highlighted those more consistently shown to modulate the cell response to a particular cytokine. The localization of the receptors has been best characterized for Fas and shown to be present in the basolateral membrane. This does not exclude expression in the apical membrane under certain circumstances. Proximal tubular cells are presented since they have been most extensively studied, but TNFSF cytokines also have actions on other tubular cells, glomerular cells, endothelial cells, leukocytes, and fibroblasts.

The FasL-Fas system participates in renal injury, regulating renal cell apoptosis and the immune and inflammatory responses [54, 59, 65]. Fas activation promotes apoptosis of cultured mesangial cells [66] and fibroblasts [18]. However, tubular cells are resistant to Fas-dependent apoptosis under basal conditions, despite the constitutive, low-level Fas expression [18, 59, 67]. Activation of these low amounts of Fas receptors results in JNK activation, not apoptosis, in renal tubular cells [68]. Other inflammatory mediators upregulating Fas are required to prime tubular cells to undergo FasL-induced apoptosis [59, 69] (Figure 1). These facts underscore the importance of the extracellular microenvironment to define cell fate in response to Fas/FasL. Renal cell FasL promotes apoptosis of lymphoid cells [59], potentially modulating the immune and inflammatory response. Consistent with novel roles as a mediator of cell stress or chronic inflammation, FasL activates NF-kappaB and the expression of proinflammatory cytokines [52, 70]. Moreover, Fas stimulation upregulates alpha(v)beta (8) integrin on tubular cells, relating Fas to cell migration and fibrosis [71].

Fas agonists induce glomerular cell apoptosis and glomerular injury characterized by proteinuria and hematuria [67]. In vivo, Fas/FasL signaling has been implicated in tubular cell apoptosis in experimental ischemic injury [72], endotoxemia [73], transplant rejection [74], chronic kidney disease [69, 75], tubulointerstitial injury of obstructive uropathy [76], and focal segmental glomerulosclerosis [77, 78]. Apoptosis of glomerular and tubular cells has also been linked to Fas/FasL expression in hypertensive renal disease [79, 80], HIV-associated nephropathy [81], and human proliferative lupus nephritis [63]. This has fueled the search for potential therapeutic applications of Fas targeting. Mice with genetically disrupted FasL/Fas systems (B6 lpr/lpr mice) or these treated with small interfering RNA targeting Fas are protected from tubular cell injury during ischemia-reperfusion [72, 82, 83] and cisplatin-induced AKI [27].

The Fas/FasL system is also a key regulator of inflammation and autoimmunity. Loss-of-function mutations on Fas (lpr/lpr) or FasL (gld/gld) on the MRL background result in lymphoproliferation, autoimmunity, and lupus-like glomerulonephritis. The autoimmune milieu appears to be the main inducer of injury, as kidney removal from the autoimmune (lpr/lpr) environment significantly reduces inflammation, and wild-type or lpr/lpr kidney grafts transplanted to lpr/lpr recipients display similar inflammation [84]. Moreover, in the course of lupus nephritis Fas deficiency does not protect from renal disease or from tubular cell apoptosis [85]. Fas and FasL may be important for resolution of inflammation, promoting apoptosis of infiltrating lymphocytes as shown in B6 lpr/lpr mice [86] and B6 gld/gld mice [87]. In addition, in FasL-defective mice (gld/gld), Fas agonists decrease renal injury, probably by limiting autoimmunity [87].

The role of Fas/FasL in renal transplantation is ambiguous: FasL gene transfer prolonged rat renal allograft survival, probably by inducting cytotoxicity in alloreactive T cells [88]. In other studies, the absence of donor kidney Fas (lpr) or FasL (gld) did not impact on histological lesions or apoptosis [58, 89] although it improved mice survival and kidney function [58].

A gene-targeted murine model exploring the relative importance of mFasL and sFasL demonstrated that mFasL is essential for cytotoxic activity, while sFasL appeared to promote autoimmunity through nonapoptotic actions, namely NF-kappaB activation. Mice that lacked sFasL (mFasL intact) appeared normal, while mice lacking mFasL (sFasL intact) had higher NF-kappaB activation and developed a lupus-like autoimmune kidney disease more severe than gld/gld mice (which lack sFasL and mFasL) [70].

## 4. TRAIL: The Saga Continues

TNF-related apoptosis-inducing ligand (TRAIL) was originally identified by two independent groups as the third member of the TNF superfamily to induce apoptosis [90, 91]. TRAIL is a type II transmembrane protein of 281 and 291 amino acids in the humans and mice, respectively, with an expected molecular mass of 33–35 kDa. Membrane-bound TRAIL can be cleaved from the cell surface to form a soluble trimeric ligand that retains the proapoptotic activity [91]. TRAIL is normally expressed in many human tissues including kidney, suggesting that TRAIL must not be cytotoxic to most tissues in vivo under normal physiological conditions [91, 92]. However, when normal cells are immersed in an inflammatory environment, data from knockout mice suggest that TRAIL may induce parenchymal cell apoptosis [93]. Two additional alternative splice variants of TRAIL in human cells lacking either exon 3 (TRAIL-beta) or exons 2 and 3 (TRAIL-gamma) had been described [94]. The lack of apoptotic activity in both isoforms and an alternative splicing in response to cytokine stimulation add complexity to the system [95].

One of the system particularities is the multiple set of TRAIL receptors. Five receptors for TRAIL have been described in humans; four membrane-bound and one soluble receptor. Of the membrane-bound receptors, TRAIL receptor 1 (TRAIL-R1, APO-2, DR4) and TRAIL receptor 2 (TRAIL-R2, DR5) contain an intact intracellular DD which is required for apoptosis induction [96]. TRAIL receptor 3 (TRAIL-R3, DcR1) has a glycosylphosphatidylinositol membrane anchor and lacks an intracellular domain, and TRAIL receptor 4 (TRAIL-R4, DcR2) contains a truncated DD. The latter may function as decoy receptors or be involved in nonapoptotic signaling [97, 98].

Osteoprotegerin is a soluble receptor without cytoplasmic or transmembrane domains, first described as a bone remodeling regulator. Osteoprotegerin is a decoy receptor for the TNFSF cytokine receptor activator of NF-kappaB ligand (RANKL) and for TRAIL [99, 100]. The affinity of TRAIL for osteoprotegerin is weaker than for transmembrane receptors [101]. However, recent studies support the biological relevance of the osteoprotegerin/TRAIL interaction in different in vitro cell systems [102–105]. Further studies to unravel the relation between TRAIL, osteoprotegerin, and RANKL could illuminate potential cross-regulatory mechanisms.

### 4.1. TRAIL and Renal Cells

 Most TRAIL literature is referred to its potent tumor cell-killing activity [106]. Different combinations of TRAIL and chemotherapeutic drugs or the use of agonistic anti-TRAILR1 or R2 antibodies shows promising results in the treatment of renal carcinoma [107, 108]. However, TRAIL also has nonapoptotic functions, such as prosurvival and proliferative effects [109–112]. In normal kidney, TRAIL is expressed only in tubules and absent from glomeruli [113]. TRAIL-R1 has a similar pattern of expression to TRAIL, while TRAIL-R2 is additionally expressed in Henle's loop [92]. TRAIL-R3 expression was not detected in the normal kidney, and there are no reports regarding renal tissue expression of TRAIL-R4. No kidney pathology has been reported in TRAIL knockout mice, suggesting that TRAIL is not required for normal kidney development and physiology [114].

### 4.2. TRAIL in Diabetic Nephropathy

 Apoptosis contributes to human DN [115]. Transcriptomics disclosed increased TRAIL and osteoprotegerin expression in human DN that correlated with parameters of kidney injury [113]. Interestingly, in DN there was *de novo* glomerular TRAIL expression and increased tubular staining. Inflammatory cytokines, such as TNF, interferon-*γ* (INF-*γ*), and macrophage migration inhibitory factor (MIF), induce TRAIL expression in tubular cells [59, 116]. A high-glucose medium, characteristic of diabetes, sensitized tubular cells and podocytes to the proapoptotic effect of TRAIL. Although it is difficult to extrapolate from cell culture studies to the in vivo situation, the low level of apoptosis induced by TRAIL in cultured tubular cells is consistent with the slow loss of renal function, over years, characteristic of DN [113]. TRAIL blockade in murine models of autoimmune diabetes (type I diabetes) led to an increased incidence and severity of disease [117–119]. Thus, depending on the type of diabetes and on the disease stage, TRAIL can have a dual role either as an immune modulator or as a regulator of renal cell survival.

## 5. The Family Grows: TWEAK

While many TNFSF ligands bind to multiple receptors [120], only a single signaling receptor for TWEAK (TWEAKR) has been confirmed [121, 122]. TWEAKR was identical to the previously characterized human fibroblast growth factor-inducible 14 (Fn14) receptor [123]. TWEAKR/Fn14 is the smallest member of the TNFRSF and lacks a DD. Initial reports that the TNFRSF protein death receptor 3 (DR3) was the TWEAK receptor [124] were not confirmed in subsequent studies [125, 126]. CD163 was recently identified as a potential scavenger receptor for TWEAK [127]. Current knowledge suggests that TWEAK and Fn14 might play a role in several processes relevant to kidney damage such as regulation of survival/proliferation of kidney cells and their ability to regenerate in response to aggression and the regulation of the inflammatory response.

### 5.1. TWEAK and Renal Cells

Both TWEAK and Fn14 are expressed by glomerular and tubular cells. The potential sources of TWEAK in the kidney include infiltrating monocytes and T lymphocytes, tubular cells, and mesangial cells [128]. Human and murine mesangial cells, podocytes, and tubular cells express Fn14 and are responsive to TWEAK [129, 130]. The process of TWEAK binding and activation of the Fn14 receptor has proliferative, proapoptotic, and proinflammatory actions in renal cells that depend on cell type and the microenvironment (Figure 1).

TWEAK, as other TNFSF members, can either induce apoptosis or proliferation depending on the experimental conditions (Figure 1). TWEAK increased the proliferation, cell number, and cyclin D1 expression of quiescent cultured tubular cells [131]. TWEAK also induced proliferation in mesangial cells and podocytes [129, 131]. TWEAK-induced tubular cell proliferation is enhanced in the presence of survival factors from serum which increase Fn14 expression [131]. There is little information about the molecular pathways that mediate TWEAK-induced proliferation. TWEAK-induced tubular cell proliferation was prevented by inhibitors of mitogen-activated protein kinases and by the NF-kappaB inhibitor parthenolide [131].

Several TNFSF cytokines, such as FasL, TNF, and TRAIL, induce apoptosis in stressed renal cells [62, 113]. Similar to FasL, TWEAK did not induce cell death in nonstimulated tubular cells. However, in the presence of inflammatory cytokines (TNF and INF*γ*), TWEAK induced apoptosis in tubular cells through the activation of the Fn14 receptor, caspases, and mitochondria involvement. TNF or INF*γ* alone increased Fn14 expression but neither was sensitized TWEAK-induced cell death. The combination of both cytokines is required to sensitize TWEAK-induced apoptosis. This, together with a more intense proliferative response, but not cell death, when Fn14 is upregulated by serum, suggests that Fn14 upregulation, per se, does not determine the type of response to TWEAK. Further, less characterized intracellular changes are required to determine the lethal or proliferative response of tubular cells to TWEAK. Interestingly, a pan-caspase inhibitor prevented TWEAK/TNF/INF*γ*-induced apoptosis, but it sensitized cells to necrosis via generation of reactive oxygen species [132].

In tubular cells TWEAK engagement of Fn14 induced a sustained NF-kappaB activation [133]. NF-kappaB activation was associated with degradation of IkappaB-alpha, nuclear translocation of RelA, and early (3–6 h) increased mRNA and protein expression of the chemokines monocyte chemotactic protein-1 (MCP-1) and RANTES. Parthenolide, which prevents IkappaB-alpha degradation, inhibited TWEAK-induced NF-kappaB activation and prevented the expression of MCP-1 and RANTES on tubular cells. TWEAK also induced the expression of inflammatory mediators in glomerular mesangial cells through NF-kappaB activation [130] and in podocytes [129].

In addition, TWEAK induces NIK-mediated, noncanonical NF-kappaB activation in tubular cells, characterized by late nuclear translocation of RelB/NF-kappaB2 DNA-binding complexes [134, 135]. The delayed TWEAK-inducted upregulation of the CCL21 and CCL19 chemokines was under noncanonical NF-kappaB control and was not observed in cells stimulated with TNF.

### 5.2. TWEAK in Renal Injury: Functional Studies

Fn14 receptor is the mediator of both the proliferative and the apoptotic effects of TWEAK, and the cell response is modulated by the cell microenvironment: in the presence of proinflammatory cytokines, TWEAK potentiates cell death while in the presence of serum TWEAK has the opposite effect, proliferation. Given the multifunctional nature of TWEAK/Fn14, only in vivo functional studies in specific diseases will clarify their role. In lupus proliferative nephritis, TWEAK/Fn14 are upregulated and TWEAK contributes to mesangial cell proliferation or apoptosis [129, 136].

TWEAK/Fn14 contribute to compensatory renal hypertrophy and hyperplasia observed following unilateral nephrectomy [131]. This is a situation characterized by tubular cell proliferation in the absence of tubular injury or increased expression of inflammatory cytokines [137]. Fn14 expression is increased in remnant kidney tubules [131]. Lower tubular cell proliferation was observed in the remnant kidney of TWEAK knockout mice compared with wild-type mice. Moreover, administration of exogenous TWEAK to uninephrectomized wild-type mice further increased renal cell proliferation [131].

AKI is characterized by renal inflammation. During AKI an initial wave of cell death is followed by compensatory tubular cell proliferation taking place in an inflammatory environment that leads to recovery. Prophylactic treatment with anti-TWEAK antibodies decreased inflammation and the rates of apoptosis and tubular cell proliferation during AKI [131, 133]. Studies with TWEAK-deficient mice confirmed a role of TWEAK in tubular cell apoptosis as well as in proliferation during AKI. These data are consistent with the proapoptotic action of TWEAK in an inflammatory milieu in cultured tubular cells [131]. Since renal function was improved by anti-TWEAK strategies and there was no delay in recovery, it was hypothesized that the reduced tubular cell proliferation during AKI observed in anti-TWEAK-treated animals reflected the lesser degree of initial injury, rather than a requirement for TWEAK for compensatory post-AKI tubular proliferation.

## 6. Conclusions

Multiple lines of evidence indicate the involvement of different TNFSF cytokines, including TNF, FasL, TRAIL, and TWEAK in the pathogenesis of renal injury. These observations may lead to the development of new therapeutic strategies. However, there is an insufficient understanding of the cooperation between cytokines in the complex in vivo environment. This information is important for the design of multipronged approaches aimed at targeting several members of the family in order to maximize benefit and minimize side effects.

## Figures and Tables

**Table 1:** TNF superfamily cytokines and receptors involved in kidney injury. Common names as well as TNFSF and TNFRSF numbers are provided.

Cytokines	Receptors		Decoy/soluble receptors	
TNF (TNFSF2)	TNFR1 (TNFRSF1A)	TNFR2 (TNFRSF1B)	sTNFR	
FasL/Apo1L/CD95L (TNFSF6)	Fas/Apo1/CD95 (TNFRSF6)		DcR3(TNFRSF6B)	
TRAIL/Apo2L (TNFSF10)	TRAILR1/DR4 (TNFRSF10A)	TRAILR2/DR5 (TNFRSF10B)	TRAILR3/DcR1 (TNFRSF10C)	TRAILR4/DcR2 (TNFRSF10D)
			Osteoprotegerin (TNFRSF11B)	
TWEAK/Apo3L (TNFSF12)	TWEAKR/Fn14 (TNFRSF12A)		CD163	

## References

[1] Aggarwal BB, Kohr WJ, Hass PE (1985). Human tumor necrosis factor: production, purification, and characterization. *The Journal of Biological Chemistry*.

[2] Pennica D, Nedwin GE, Hayflick JS (1984). Human tumour necrosis factor: precursor structure, expression and homology to lymphotoxin. *Nature*.

[3] Gruss H-J, Dower SK (1995). Tumor necrosis factor ligand superfamily: involvement in the pathology of malignant lymphomas. *Blood*.

[4] Bodmer J-L, Schneider P, Tschopp J (2002). The molecular architecture of the TNF superfamily. *Trends in Biochemical Sciences*.

[5] Aggarwal BB (2003). Signalling pathways of the TNF superfamily: a double-edged sword. *Nature Reviews Immunology*.

[6] Lotz M, Setareh M, von Kempis J, Schwarz H (1996). The nerve growth factor/tumor necrosis factor receptor family. *Journal of Leukocyte Biology*.

[7] Armitage RJ (1994). Tumor necrosis factor receptor superfamily members and their ligands. *Current Opinion in Immunology*.

[8] Cosman D (1994). A family of ligands for the TNF receptor superfamily. *Stem Cells*.

[9] Hehlgans T, Pfeffer K (2005). The intriguing biology of the tumour necrosis factor/tumour necrosis factor receptor superfamily: players, rules and the games. *Immunology*.

[10] Foster D, Parrish-Novak J, Fox B, Xu W (2004). Cytokine-receptor pairing: accelerating discovery of cytokine function. *Nature Reviews Drug Discovery*.

[11] Tansey MG, Szymkowski DE (2009). The TNF superfamily in 2009: new pathways, new indications, and new drugs. *Drug Discovery Today*.

[12] Sanz AB, Sanchez-Niño MD, Ramos AM (2010). NF-*κ*B in renal inflammation. *Journal of the American Society of Nephrology*.

[13] Mas S, Martínez-Pinna R, Martín-Ventura JL (2010). Local non-esterified fatty acids correlate with inflammation in atheroma plaques of patients with type 2 diabetes. *Diabetes*.

[14] Ortiz A, Egido J (1995). Is there a role for specific anti-TNF strategies in glomerular diseases?. *Nephrology Dialysis Transplantation*.

[15] Ernandez T, Mayadas TN (2009). Immunoregulatory role of TNF*α* in inflammatory kidney diseases. *Kidney International*.

[16] MacEwan DJ (2002). TNF receptor subtype signalling: differences and cellular consequences. *Cellular Signalling*.

[17] Gresser I, Woodrow D, Moss J (1987). Toxic effects of recombinant tumor necrosis factor in suckling mice: comparisons with interferon *α*/*β*. *American Journal of Pathology*.

[18] Ortiz A, Lorz C, González-Cuadrado S, Garcia Del Moral R, O’Valle F, Egido J (1997). Cytokines and Fas regulate apoptosis in murine renal interstitial fibroblasts. *Journal of the American Society of Nephrology*.

[19] Misseri R, Meldrum DR, Dinarello CA (2005). TNF-*α* mediates obstruction-induced renal tubular cell apoptosis and proapoptotic signaling. *American Journal of Physiology*.

[20] Donnahoo KK, Shames BD, Harken AH, Meldrum DR (1999). The role of tumor necrosis factor in renal ischemia-reperfusion injury. *Journal of Urology*.

[21] Navarro JF, Mora-Fernández C (2006). The role of TNF-*α* in diabetic nephropathy: pathogenic and therapeutic implications. *Cytokine and Growth Factor Reviews*.

[22] Khan SB, Cook HT, Bhangal G, Smith J, Tam FWK, Pusey CD (2005). Antibody blockade of TNF-*α* reduces inflammation and scarring in experimental crescentic glomerulonephritis. *Kidney International*.

[23] Egido J, Gomez-Chiarri M, Ortiz A (1993). Role of tumor necrosis factor-*α* in the pathogenesis of glomerular diseases. *Kidney International, Supplement*.

[24] Ramesh G, Brian Reeves W (2002). TNF-*α* mediates chemokine and cytokine expression and renal injury in cisplatin nephrotoxicity. *The Journal of Clinical Investigation*.

[25] Daemen MARC, van de Ven MWCM, Heineman E, Buurman WA (1999). Involvement of endogenous interleukin-10 and tumor necrosis factor-*α* in renal, ischemia-reperfusion injury. *Transplantation*.

[26] Al-Lamki RS, Wang J, Vandenabeele P (2005). TNFR1- and TNFR2-mediated signaling pathways in human kidney are cell type-specific and differentially contribute to renal injury. *The FASEB Journal*.

[27] Tsuruya K, Ninomiya T, Tokumoto M (2003). Direct involvement of the receptor-mediated apoptotic pathways in cisplatin-induced renal tubular cell death. *Kidney International*.

[28] Ramesh G, Reeves WB (2003). TNFR2-mediated apoptosis and necrosis in cisplatin-induced acute renal failure. *American Journal of Physiology*.

[29] Guo G, Morrissey J, McCracken R, Tolley T, Klahr S (1999). Role of TNFR1 and TNFR2 receptors in tubulointerstitial fibrosis of obstructive nephropathy. *American Journal of Physiology*.

[30] Zhou T, Edwards CK, Yang P, Wang Z, Bluethmann H, Mountz JD (1996). Greatly accelerated lymphadenopathy and autoimmune disease in lpr mice lacking tumor necrosis factor receptor I. *The Journal of Immunology*.

[31] Vielhauer V, Stavrakis G, Mayadas TN (2005). Renal cell-expressed TNF receptor 2, not receptor 1, is essential for the development of glomerulonephritis. *The Journal of Clinical Investigation*.

[32] Haridas V, Darnay BG, Natarajan K, Heller R, Aggarwal BB (1998). Overexpression of the p80 TNF receptor leads to TNF-dependent apoptosis, nuclear factor-*κ*B activation, and c-Jun kinase activation. *The Journal of Immunology*.

[33] Aringer M, Houssiau F, Gordon C (2009). Adverse events and efficacy of TNF-*α* blockade with infliximab in patients with systemic lupus erythematosus: long-term follow-up of 13 patients. *Rheumatology*.

[34] Aringer M, Smolen JS (2008). Efficacy and safety of TNF-blocker therapy in systemic lupus erythematosus. *Expert Opinion on Drug Safety*.

[35] Booth A, Harper L, Hammad T (2004). Prospective study of TNF*α* blockade with infliximab in anti-neutrophil cytoplasmic antibody-associated systemic vasculitis. *Journal of the American Society of Nephrology*.

[36] Lamprecht P, Voswinkel J, Lilienthal T (2002). Effectiveness of TNF-*α* blockade with infliximab in refractory Wegener’s granulomatosis. *Rheumatology*.

[37] Morgan MD, Drayson MT, Savage COS, Harper L (2010). Addition of infliximab to standard therapy for ANCA-associated Vasculitis. *Nephron—Clinical Practice*.

[38] Laurino S, Chaudhry A, Booth A, Conte G, Jayne D (2010). Prospective study of TNF*α* blockade with adalimumab in ANCA-associated systemic vasculitis with renal involvement. *Nephrology Dialysis Transplantation*.

[39] Feldmann M, Pusey CD (2006). Is there a role for TNF-*α* in anti-neutrophil cytoplasmic antibody-associated vasculitis? Lessons from other chronic inflammatory diseases. *Journal of the American Society of Nephrology*.

[40] Huugen D, Cohen Tervaert JW, Heeringa P (2006). TNF-*α* bioactivity-inhibiting therapy in ANCA-associated vasculitis: clinical and experimental considerations. *Clinical Journal of the American Society of Nephrology*.

[41] Charles PJ, Smeenk RJT, De Jong J, Feldmann M, Maini RN (2000). Assessment of antibodies to double-stranded DNA induced in rheumatoid arthritis patients following treatment with infliximab, a monoclonal antibody to tumor necrosis factor *α*: findings in open-label and randomized placebo-controlled trials. *Arthritis and Rheumatism*.

[42] De Bandt M, Sibilia J, Le Loët X (2005). Systemic lupus erythematosus induced by anti-tumour necrosis factor alpha therapy: a French national survey. *Arthritis Research &amp; Therapy*.

[43] Crum NF, Lederman ER, Wallace MR (2005). Infections associated with tumor necrosis factor-*α* antagonists. *Medicine*.

[44] Saliu OY, Sofer C, Stein DS, Schwander SK, Wallis RS (2006). Tumor-necrosis-factor blockers: differential effects on mycobacterial immunity. *Journal of Infectious Diseases*.

[45] Nagata S, Golstein P (1995). The Fas death factor. *Science*.

[46] Trambas CM, Griffiths GM (2003). Delivering the kiss of death. *Nature Immunology*.

[47] Schulte M, Reiss K, Lettau M (2007). ADAM10 regulates FasL cell surface expression and modulates FasL-induced cytotoxicity and activation-induced cell death. *Cell Death and Differentiation*.

[48] Ashkenazi A, Dixit VM (1999). Apoptosis control by death and decoy receptors. *Current Opinion in Cell Biology*.

[49] Suda T, Hashimoto H, Tanaka M, Ochi T, Nagata S (1997). Membrane Fas ligand kills human peripheral blood T lymphocytes, and soluble fas ligand blocks the killing. *Journal of Experimental Medicine*.

[50] Schneider P, Holler N, Bodmer J-L (1998). Conversion of membrane-bound Fas(CD95) ligand to its soluble form is associated with downregulation of its proapoptotic activity and loss of liver toxicity. *Journal of Experimental Medicine*.

[51] Strasser A, Jost PJ, Nagata S (2009). The Many Roles of FAS Receptor Signaling in the Immune System. *Immunity*.

[52] Peter ME, Budd RC, Desbarats J (2007). The CD95 receptor: apoptosis revisited. *Cell*.

[53] Miwa K, Asano M, Horai R, Iwakura Y, Nagata S, Suda T (1998). Caspase 1-independent IL-1*β* release and inflammation induced by the apoptosis inducer Fas ligand. *Nature Medicine*.

[54] Ortiz A, Lorz C, Egido J (1999). The Fas ligand/Fas system in renal injury. *Nephrology Dialysis Transplantation*.

[55] Ross MJ, Martinka S, D’Agati VD, Bruggeman LA (2005). NF-*κ*B regulates Fas-mediated apoptosis in HIV-associated nephropathy. *Journal of the American Society of Nephrology*.

[56] Eichler T, Ma Q, Kelly C (2006). Single and combination toxic metal exposures induce apoptosis in cultured murine podocytes exclusively via the extrinsic caspase 8 pathway. *Toxicological Sciences*.

[57] Wang C, Peng H, Tang H (2007). Serum IgA1 from IgA nephropathy patients induces apoptosis in podocytes through direct and indirect pathways. *Clinical and Investigative Medicine*.

[58] Du C, Jiang J, Guan Q (2004). Renal tubular epithelial cell self-injury through Fas/Fas ligand interaction promotes renal allograft injury. *American Journal of Transplantation*.

[59] Lorz C, Ortiz A, Justo P (2000). Proapoptotic Fas ligand is expressed by normal kidney tubular epithelium and injured glomeruli. *Journal of the American Society of Nephrology*.

[60] Lorz C, Justo P, Sanz A, Subirá D, Egido J, Ortiz A (2004). Paracetamol-induced renal tubular injury: a role for ER stress. *Journal of the American Society of Nephrology*.

[61] Justo P, Lorz C, Sanz A, Egido J, Ortiz A (2003). Intracellular Mechanisms of Cyclosporin A-Induced Tubular Cell Apoptosis. *Journal of the American Society of Nephrology*.

[62] Ortiz A, Lorz C, Egido J (1999). New kids in the block: the role of FasL and Fas in kidney damage. *Journal of Nephrology*.

[63] Tsukinoki T, Sugiyama H, Sunami R (2004). Mesangial cell Fas ligand: upregulation in human lupus nephritis and NF-*κ*B-mediated expression in cultured human mesangial cells. *Clinical and Experimental Nephrology*.

[64] Tan KH, Hunziker W (2003). Compartmentalization of Fas and Fas ligand may prevent auto- or paracrine apoptosis in epithelial cells. *Experimental Cell Research*.

[65] Boonstra JG, van der Woude FJ, Wever PC, Laterveer JC, Daha MR, van Kooten C (1997). Expression and function of Fas (CD95) on human renal tubular epithelial cells. *Journal of the American Society of Nephrology*.

[66] González-Cuadrado S, López-Armada M-J, Gómez-Guerrero C (1996). Anti-Fas antibodies induce cytolysis and apoptosis in cultured human mesangial cells. *Kidney International*.

[67] Gonzalez-Cuadrado S, Lorz C, García Del Moral R (1997). Agonistic anti-Fas antibodies induce glomerular cell apoptosis in mice in vivo. *Kidney International*.

[68] Khan S, Koepke A, Jarad G (2001). Apoptosis and JNK activation are differentially regulated by Fas expression level in renal tubular epithelial cells. *Kidney International*.

[69] Schelling JR, Nkemere N, Kopp JB, Cleveland RP (1998). Fas-dependent fratricidal apoptosis is a mechanism of tubular epithelial cell deletion in chronic renal failure. *Laboratory Investigation*.

[70] O Reilly LA, Tai L, Lee L (2009). Membrane-bound Fas ligand only is essential for Fas-induced apoptosis. *Nature*.

[71] Jarad G, Wang B, Khan S (2002). Fas activation induces renal tubular epithelial cell ß8 integrin expression and function in the absence of apoptosis. *The Journal of Biological Chemistry*.

[72] Nogae S, Miyazaki M, Kobayashi N (1998). Induction of apoptosis in ischemia-reperfusion model of mouse kidney: possible involvement of Fas. *Journal of the American Society of Nephrology*.

[73] Ortiz-Arduan A (1996). Regulation of Fas and Fas ligand expression in cultured murine renal cells and in the kidney during endotoxemia. *American Journal of Physiology*.

[74] Matsuno T, Sasaki H, Nakagawa K (1998). Expression of Fas/Fas ligand and apoptosis induction during renal allograft rejection. *Transplantation Proceedings*.

[75] Schelling JR, Cleveland RP (1999). Involvement of Fas-dependent apoptosis in renal tubular epithelial cell deletion in chronic renal failure. *Kidney International*.

[76] Hoi Y-J, Baranowska-Daca E, Nguyen V (2000). Mechanism of chronic obstructive uropathy: increased expression of apoptosis-promoting molecules. *Kidney International*.

[77] Wang W, Tzanidis A, Divjak M, Thomson NM, Stein-Oakley AN (2001). Altered signaling and regulatory mechanisms of apoptosis in focal and segmental glomerulosclerosis. *Journal of the American Society of Nephrology*.

[78] Erkan E, Garcia CD, Patterson LT (2005). Induction of renal tubular cell apoptosis in focal segmental glomerulosclerosis: roles of proteinuria and Fas-dependent pathways. *Journal of the American Society of Nephrology*.

[79] Sanders PW, Wang P-X (2002). Activation of the Fas/Fas ligand pathway in hypertensive renal disease in Dahl/Rapp rats. *BMC Nephrology*.

[80] Ying W-Z, Wang P-X, Sanders PW (2000). Induction of apoptosis during development of hypertensive nephrosclerosis. *Kidney International*.

[81] Kelly JG, Carpenter RN, Tague JA (1992). Object classification and acoustic imaging with active sonar. *Journal of the Acoustical Society of America*.

[82] Hamar P, Song E, Kökeny G, Chen A, Ouyang N, Lieberman J (2004). Small interfering RNA targeting Fas protects mice against renal ischemia-reperfusion injury. *Proceedings of the National Academy of Sciences of the United States of America*.

[83] Du C, Wang S, Diao H, Guan Q, Zhong R, Jevnikar AM (2006). Increasing resistance of tubular epithelial cells to apoptosis by shRNA therapy ameliorates renal ischemia-reperfusion injury. *American Journal of Transplantation*.

[84] Hamar P, Wang M, Godó M (2010). Lupus nephritis reoccurs following transplantation in the lupus prone mouse. *Lupus*.

[85] Wada T, Schwarting A, Kinoshita K (1999). Fas on renal parenchymal cells does not promote autoimmune nephritis in MRL mice. *Kidney International*.

[86] Fleck M, Kern ER, Zhou T (1998). Apoptosis mediated by Fas but not tumor necrosis factor receptor 1 prevents chronic disease in mice infected with murine cytomegalovirus. *The Journal of Clinical Investigation*.

[87] Zhang H-G, Fleck M, Kern ER (2000). Antigen presenting cells expressing Fas ligand down-modulate chronic inflammatory disease in Fas ligand-deficient mice. *The Journal of Clinical Investigation*.

[88] Swenson KM, Bibo KE, Wang T (1998). Fas ligand gene transfer to renal allografts in rats: effects on allograft survival. *Transplantation*.

[89] Kayser D, Einecke G, Famulski KS (2008). Donor Fas is not necessary for T-cell-mediated rejection of mouse kidney allografts. *American Journal of Transplantation*.

[90] Wiley SR, Schooley K, Smolak PJ (1995). Identification and characterization of a new member of the TNF family that induces apoptosis. *Immunity*.

[91] Pitti RM, Marsters SA, Ruppert S, Donahue CJ, Moore A, Ashkenazi A (1996). Induction of apoptosis by Apo-2 ligand, a new member of the tumor necrosis factor cytokine family. *The Journal of Biological Chemistry*.

[92] Spierings DC, de Vries EG, Vellenga E (2004). Tissue distribution of the death ligand TRAIL and its receptors. *Journal of Histochemistry and Cytochemistry*.

[93] Zheng S-J, Wang P, Tsabary G, Chen YH (2004). Critical roles of TRAIL in hepatic cell death and hepatic inflammation. *The Journal of Clinical Investigation*.

[94] Krieg A, Krieg T, Wenzel M (2003). TRAIL-*β* and TRAIL-*γ* two novel splice variants of the human TNF-related apoptosis-inducing ligand (TRAIL) without apoptotic potential. *British Journal of Cancer*.

[95] Kamachi M, Aramaki T, Tanimura S (2007). Activation of protein phosphatase causes alternative splicing of tumor necrosis factor-related apoptosis-inducing ligand (TRAIL): potential effect on immune surveillance. *Biochemical and Biophysical Research Communications*.

[96] MacFarlane M, Ahmad M, Srinivasula SM, Fernandes-Alnemri T, Cohen GM, Alnemri ES (1997). Identification and molecular cloning of two novel receptors for the cytotoxic ligand TRAIL. *The Journal of Biological Chemistry*.

[97] Zhang XD, Franco AV, Nguyen T, Gray CP, Hersey P (2000). Differential localization and regulation of death and decoy receptors for TNF-related apoptosis-inducing ligand (TRAIL) in human melanoma cells. *The Journal of Immunology*.

[98] Rimondi E, Secchiero P, Quaroni A, Zerbinati C, Capitani S, Zauli G (2006). Involvement of TRAIL/TRAIL-receptors in human intestinal cell differentiation. *Journal of Cellular Physiology*.

[99] Emery JG, McDonnell P, Burke MB (1998). Osteoprotegerin is a receptor for the cytotoxic ligand TRAIL. *The Journal of Biological Chemistry*.

[100] Simonet WS, Lacey DL, Dunstan CR (1997). Osteoprotegerin: a novel secreted protein involved in the regulation of bone density. *Cell*.

[101] Truneh A, Sharma S, Silverman C (2000). Temperature-sensitive differential affinity of TRAIL for its receptors: DR5 is the highest affinity receptor. *The Journal of Biological Chemistry*.

[102] Holen I, Croucher PI, Hamdy FC, Eaton CL (2002). Osteoprotegerin (OPG) is a survival factor for human prostate cancer cells. *Cancer Research*.

[103] Pritzker LB, Scatena M, Giachelli CM (2004). The role of osteoprotegerin and tumor necrosis factor-related apoptosis-inducing ligand in human microvascular endothelial cell survival. *Molecular Biology of the Cell*.

[104] Miyashita T, Kawakami A, Nakashima T (2004). Osteoprotegerin (OPG) acts as an endogenous decoy receptor in tumour necrosis factor-related apoptosis-inducing ligand (TRAIL)-mediated apoptosis of fibroblast-like synovial cells. *Clinical and Experimental Immunology*.

[105] Shipman CM, Croucher PI (2003). Osteoprotegerin is a soluble decoy receptor for tumor necrosis factor-related apoptosis-inducing ligand/Apo2 ligand and can function as a paracrine survival factor for human myeloma cells. *Cancer Research*.

[106] Walczak H, Miller RE, Ariail K (1999). Tumoricidal activity of tumor necrosis factor-related apoptosis-inducing ligand in vivo. *Nature Medicine*.

[107] Wu X-X, Ogawa O, Kakehi Y (2004). TRAIL and chemotherapeutic drugs in cancer therapy. *Vitamins and Hormones*.

[108] Marini P (2006). Drug evaluation: lexatumumab, an intravenous human agonistic mAb targeting TRAIL receptor 2. *Current Opinion in Molecular Therapeutics*.

[109] Di Pietro R, Zauli G (2004). Emerging non-apoptotic functions of tumor necrosis factor-related apoptosis-inducing ligand (TRAIL)/Apo2L. *Journal of Cellular Physiology*.

[110] Chu Z-L, McKinsey TA, Liu L, Gentry JJ, Malim MH, Ballard DW (1997). Suppression of tumor necrosis factor-induced cell death by inhibitor of apoptosis c-IAP2 is under NF-*κ*B control. *Proceedings of the National Academy of Sciences of the United States of America*.

[111] Secchiero P, Melloni E, Heikinheimo M (2004). TRAIL regulates normal erythroid maturation through an ERK-dependent pathway. *Blood*.

[112] Secchiero P, Zerbinati C, Rimondi E (2004). TRAIL promotes the survival, migration and proliferation of vascular smooth muscle cells. *Cellular and Molecular Life Sciences*.

[113] Lorz C, Benito-Martin A, Boucherot A (2008). The death ligand TRAIL in diabetic nephropathy. *Journal of the American Society of Nephrology*.

[114] Cretney E, Takeda K, Yagita H, Glaccum M, Peschon JJ, Smyth MJ (2002). Increased susceptibility to tumor initiation and metastasis in TNF-related apoptosis-inducing ligand-deficient mice. *The Journal of Immunology*.

[115] Kumar D, Robertson S, Burns KD (2004). Evidence of apoptosis in human diabetic kidney. *Molecular and Cellular Biochemistry*.

[116] Sanchez-Niño MD, Sanz AB, Ihalmo P (2009). The MIF receptor CD74 in diabetic podocyte injury. *Journal of the American Society of Nephrology*.

[117] Lamhamedi-Cherradi S-E, Zheng S-J, Maguschak KA, Peschon J, Chen YH (2003). Defective thymocyte apoptosis and accelerated autoimmune diseases in TRAIL-/- mice. *Nature Immunology*.

[118] Lamhamedi-Cherradi S-E, Zheng S, Tisch RM, Chen YH (2003). Critical roles of tumor necrosis factor-related apoptosis-inducing ligand in type 1 diabetes. *Diabetes*.

[119] Mi Q-S, Ly D, Lamhamedi-Cherradi S-E (2003). Blockade of tumor necrosis factor-related apoptosis-inducing ligand exacerbates type 1 diabetes in NOD mice. *Diabetes*.

[120] Bossen C, Ingold K, Tardivel A (2006). Interactions of tumor necrosis factor (TNF) and TNF receptor family members in the mouse and human. *The Journal of Biological Chemistry*.

[121] Nakayama M, Ishidoh K, Kojima Y (2003). Fibroblast growth factor-inducible 14 mediates multiple pathways of TWEAK-induced cell death. *The Journal of Immunology*.

[122] Wiley SR, Winkles JA (2003). TWEAK, a member of the TNF superfamily, is a multifunctional cytokine that binds the TweakR/Fn14 receptor. *Cytokine and Growth Factor Reviews*.

[123] Meighan-Mantha RL, Hsu DKW, Guo Y (1999). The mitogen-inducible Fn14 gene encodes a type I transmembrane protein that modulates fibroblast adhesion and migration. *The Journal of Biological Chemistry*.

[124] Marsters SA, Sheridan JP, Pitti RM, Brush J, Goddard A, Ashkenazi A (1998). Identification of a ligand for the death-domain-containing receptor Apo3. *Current Biology*.

[125] Kaptein A, Jansen M, Dilaver G (2000). Studies on the interaction between TWEAK and the death receptor WSL-1/TRAMP (DR3). *FEBS Letters*.

[126] Nakayama M, Ishidoh K, Kayagaki N (2002). Multiple pathways of TWEAK-induced cell death. *The Journal of Immunology*.

[127] Bover LC, Cardó-Vila M, Kuniyasu A (2007). A previously unrecognized protein-protein interaction between TWEAK and CD163: potential biological implications. *The Journal of Immunology*.

[128] Ortiz A, Sanz AB, García BM (2009). Considering TWEAK as a target for therapy in renal and vascular injury. *Cytokine and Growth Factor Reviews*.

[129] Gao H-X, Campbell SR, Burkly LC (2009). TNF-like weak inducer of apoptosis (TWEAK) induces inflammatory and proliferative effects in human kidney cells. *Cytokine*.

[130] Campbell S, Burkly LC, Gao H-X (2006). Proinflammatory effects of Tweak/Fn14 interactions in glomerular mesangial cells. *The Journal of Immunology*.

[131] Sanz AB, Sanchez-Niño MD, Izquierdo MC (2009). Tweak induces proliferation in renal tubular epithelium: a role in uninephrectomy induced renal hyperplasia. *Journal of Cellular and Molecular Medicine*.

[132] Justo P, Sanz AB, Sanchez-Niño MD (2006). Cytokine cooperation in renal tubular cell injury: the role of TWEAK. *Kidney International*.

[133] Sanz AB, Justo P, Sanchez-Nino MD (2008). The cytokine TWEAK modulates renal tubulointerstitial inflammation. *Journal of the American Society of Nephrology*.

[134] Sanz AB, Sanchez-Niño MD, Izquierdo MC (2010). TWEAK activates the non-canonical NF*κ*B pathway in murine renal tubular cells: modulation of CCL21. *PLoS ONE*.

[135] Sanz AB, Santamaría B, Ruiz-Ortega M, Egido J, Ortiz A (2008). Mechanisms of renal apoptosis in health and disease. *Journal of the American Society of Nephrology*.

[136] Molano A, Lakhani P, Aran A, Burkly LC, Michaelson JS, Putterman C (2009). TWEAK stimulation of kidney resident cells in the pathogenesis of graft versus host induced lupus nephritis. *Immunology Letters*.

[137] Sun J, Langer WJ, Devish K, Lane PH (2006). Compensatory kidney growth in estrogen receptor-*α* null mice. *American Journal of Physiology*.

